# Improvement of the Machining Performance of the TW-ECDM Process Using Magnetohydrodynamics (MHD) on Quartz Material

**DOI:** 10.3390/ma14092377

**Published:** 2021-05-03

**Authors:** Ankit D. Oza, Abhishek Kumar, Vishvesh Badheka, Amit Arora, Manoj Kumar, Catalin I. Pruncu, Tej Singh

**Affiliations:** 1 Industrial Engineering Department, Pandit Deendayal Energy University, Gandhinagar 382007, India; abhishek.K@sot.pdpu.ac.in; 2 Mechanical Engineering Department, Pandit Deendayal Energy University, Gandhinagar 382007, India; vishvesh.Badheka@spt.pdpu.ac.in; 3 Materials Engineering, Indian Institute of Technology, Gandhinagar-382355, India; amitarora@iitgn.ac.in; 4 Production & Industrial Engineering Department, Punjab Engineering College, Chandigarh 160012, India; mnjbrd.02@gmail.com; 5 Mechanical Engineering, Imperial College London, Exhibition Rd., London SW7 2AZ, UK; 6 Design, Manufacturing & Engineering Management, University of Strathclyde, Glasgow G1 1XJ, UK; 7 Savaria Institute of Technology, ELTE Eötvös Loránd University, 9700 Szombathely, Hungary; sht@inf.elte.hu

**Keywords:** electrolyte, machining, microslits, MHD, MRR, roughness

## Abstract

Many microslits are typically manufactured on quartz substrates and are used to improve their industrial performance. The fabrication of microslits on quartz is difficult and expensive to achieve using recent traditional machining processes due to its hardness, electrically insulating nature, and brittleness. The key objective of the current study was to demonstrate the fabrication of microslits on quartz material through a magnetohydrodynamics (MHD)-assisted traveling wire-electrochemical discharge micromachining process. Hydrogen gas bubbles were concentrated around the entire wire surface during electrolysis. This led to a less active dynamic region of the wire electrode, which decreased the adequacy of the electrolysis process and the machining effectiveness. The test results affirmed that the MHD convection approach evacuated the gas bubbles more rapidly and improved the void fraction in the gas bubble scattering layer. Furthermore, the improvements in the material removal rate and length of the cut were 85.28% and 48.86%, respectively, and the surface roughness was reduced by 30.39% using the MHD approach. A crossover methodology with a Taguchi design and ANOVA was utilized to study the machining performance. This exploratory investigation gives an unused strategy that shows a few advantages over the traditional TW-ECDM process.

## 1. Introduction

In recent times, the usage of miniature products and their applications has significantly increased. This has led to an increase in the demand to create complex geometries with precise dimensions and high quality. These devices are comprehensively studied, which allows for constructive applications for chemical and biological investigation [1]. Generally, microchannels/slits are made of glass and polymer materials [2]. The microchannel chips that are made from polymeric materials have many limitations, viz. low solidity after exposure to a heated atmosphere, meager chemical resistance and optical properties, and low porousness to moisture [3,4]. Therefore, quantifiable fabrication methods have been examined for quartz-glass-based microchannels [5]. The patterns created in glass and ceramics are difficult to create due to the brittleness of quartz glass. This led to glass and ceramics being replaced by polydimethylsiloxane (PDMS) in many applications [6]. Quartz glass may be an immaculate shape of silica with a SiO_2_ content of more than 99.9% and it has been used widely in lenses, computers, resonators, windows, smartphones, GPS equipment, communication (3C) engineering, and electronic industries for high-quality tuned circuits. Quartz is widely used in micro device testing, metrology components, jewelry, gemstones, frequency control, mirror substrates, piezoelectric applications for converting electrical to mechanical energy (and vice versa), and medical incision devices [6,7,8,9]. Owing to its higher hardness, brittleness, and higher melting point, machining quartz is difficult [10] and thus quartz was used as the workpiece material for the study.

The literature reveals that microslits on quartz material can be fabricated using various unconventional machining processes. Overcut and rough surfaces were observed with ultrashort laser pulse processes [11]. When using a Nd:YAG laser, damage and thermal cracks on the the inner machine surface were observed [12,13]. When an abrasive slurry jet was applied to machine the quartz, a large wavy pattern was observed and the material removal rate decreased [14]. Microedge cracks and burrs were noticed when a laser-assisted dry microgrinding process was applied [15]. Burrs can be reduced using laser-assisted dry microgrinding machining processes but the heat affected zone (HAZ) across the surface is a challenge [16]. When powder-mixed rotary ultrasonic motion was applied to quartz, the material removal rate increased but the chances of crack propagation limits its use [17]. Rounded cracks were formed and the machined surface melted when using the electrochemical spark trepanning process [18]. A high aspect ratio was obtained using the ultra-short laser-machining processes; however, the surface roughness and surface quality were poor [19]. Furthermore, these processes had a high setup and machining cost. Other developed and broadly acknowledged machining processes, such as electrochemical machining (ECM) and wire electrical discharge machining (W-EDM), are only able to machine electrically conducting materials [20,21,22]. To overcome the previously stated key impediments, there emerges a need to create an advanced hybrid machining process that can machine different electrically nonconducting materials and make strides in the quality and nature of the machining performance [23,24].

**Figure 1:**
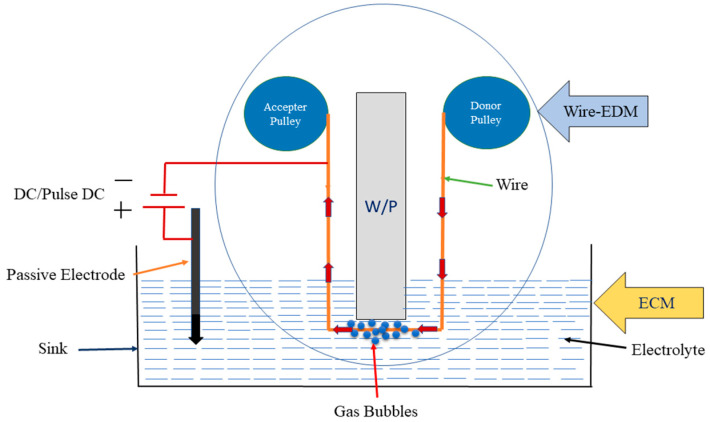
The TW-ECDM process [20].

TW-ECDM is an eminent micromachining approach with enormous potential for machining (slicing/cutting) two-dimensional and complicated profiles of a wide variety of hard, thermal-shock-resistant, indispensable, brittle, and electrically insulating materials, such as glass, zirconia, alumina, and composites. It is also known as the traveling wire electrochemical spark machining (TW-ECSM) process. A TW-ECDM process is a hybrid approach of the ECM and W-EDM processes that offers a 5 to 50 times higher material removal rate than W-EDM and ECM, and it diminishes the tool wear rate [25]. The TW-ECDM process is steadier than W-EDM and gives a better surface finish when contrasted with ECM. In the TW-ECDM process, both the electric discharge erosion and electrochemical dissolution (ESD) impacts are utilized for the evacuation of the material [26]. This helps to machine the workpiece and electrochemical dissolution gives a better and smoother surface finish. Figure 1 schematically demonstrates the TW-ECDM process. In this procedure, two terminals (electrodes) are utilized, which are plunged into the electrolyte. The electrolyte could be either alkaline or acidic. A traveling (circulating) wire is used as a cathode, which is used to cut or slice the workpiece and the auxiliary (assistant or passive) cathode is utilized as an anode. A circulating wire is kept away (0.1–0.5 mm) from the workpiece and the passive electrode is kept around 30–80 mm away from the machining zone to preserve the current density. With an increase in DC power, electrolysis is observed, and further increasing the voltage results in gas bubble generation, which is responsible for generating the spark [27]. It was observed that gas bubble formation increases with the applied voltage that was responsible for generating the spark. By further increasing the voltage, the bubbles begin to break down and provide a steady gas film around the wire. If the potential difference is high enough for a given pair of electrodes, sparking is observed, where this spark causes a streamline of high-energy electrons that are transferred with exceptionally high speed toward the workpiece. This burst of electrons produces compressive shock waves on the surface of the workpiece, which results in removing the material via melting, vaporization, and thermal erosion [28,29,30].

Many researchers have studied various aspects of the TW-ECDM process. Table 1 represents the various wires (tool electrodes) and their sizes, workpiece materials, and key findings regarding the TW-ECDM process till now. The use of a pulse DC supply reduced the surface roughness as compared to simple DC, and therefore, pulse a DC supply was selected for the present study [31]. The machining performance was improved by keeping a minimum distance between the wire and the workpiece [32]. Thermal cracks, the HAZ, and a high kerf width were found for a higher voltage, which reduced the precision and quality of machined slits, and therefore, it needs to be minimized. The electric field through the tool electrode (wire) and electrolyte was found to be responsible for producing the spark. A titrated flow for the electrolyte in the TW-ECDM process was proposed to improve the gas film formation, quality of the machined slits, and reduce the waste of the electrolyte [33]. It flushed the burrs from the cutting area and produced a stable gas film; as a result, a long and straight slit with a minimum kerf width was observed. The use of controlled electrolyte flow enhanced the machining efficiency and quality at a lesser cost. The addition of SiC particles with the titrated electrolytic flow was explored and, as a result, the surface texture improved by 80% and the mean slit breadth reduced from 200 to 185 μm [34].

The combined effects of the sparking mechanism, electrolysis, and abrasive cutting on the surface roughness, slit expansion, and MRR were reported. It was noticed that the reciprocating mechanism in TW-ECDM helped to machine the irregular cross-section [36]. Furthermore, by adding abrasive particles into the electrolyte, the slit expansion was reduced because the abrasive particles disturbed the accumulated gas bubbles that formed an isolating film around the wire. To minimize the slit expansion, the duty factor should be low and the power frequency should be high. Therefore, during the present study, to reduce the slit expansion/kerf width, a low duty factor and a high power frequency were selected.

During machining, when the wire is in contact with the electrolyte, a high electric field intensity occurs at that spot; as a result, wire breakage occurs. Another reason for frequent wire breakage is the breakdown of the gas film [28]. Discharge current flows in KOH for less time as compared to NaOH. Furthermore, the impulsive force is lower, and therefore, the slit expansion is reduced [36]; as such, in the present study, a KOH electrolyte was used. Recent studies of the W-EDM process showed that the use of a coated wire in W-EDM gives better machining performance and improves the strength, conductivity, flushability, cutting speed, cooling, and sparking abilities [47]. A coated wire runs smoothly without rupturing in comparison to a conventional wire, and consequently, a zinc-coated brass wire was chosen because of its cooling, flushability, and cutting speed abilities [48].

All the prior detailed research regarding this topic is restricted to the fabrication of microslits on quartz and it is clear that a need still exists for an electrolyte and a wire that can efficiently machine microslits. During the electrolysis process, bubbles form and stick on the surface of the wire (tool electrode) after a less active surface area of wire becomes available, which reduces the efficiency of the electrolysis process. Furthermore, bombarding electrons raise the temperature of the workpiece for a short time, and due to the quenching phenomenon, the temperature of the workpiece decreases; thus, better electrolysis is required for stable machining. Moreover, for stable and efficient machining performance, it is required to reduce the wire breakage, improve the gas film formation, eliminating the debris (fine size) from the machining area, and provide a uniform electrolysis process. For this reason, a stable and efficient machining process, along with a better electrolysis process, are crucial determinants of the quartz machining quality and merit further analysis. The MHD approach can be a potential solution as it improves the electrolysis process and flushes debris from the machining area. To produce stable machining operations, the wire breakage phenomenon needs to be minimized, and thus, a zinc-coated brass wire was used to reduce the wire breakage during the process. Therefore, in the present work, an experiment was undertaken to fabricate microslits on quartz material with the integration of MHD-assisted TW-ECDM and a zinc-coated brass wire approach. The applied voltage, percentage of electrolyte concentration, and wire speed were selected as the input process parameters, and the material removal rate (MRR), length of cut (LOC), and surface roughness (R_a_) were selected as the quality characteristics. The subsequent section illustrates the materials and methods that were used to conduct the investigation.

## 2. Experimental Methodology

**Figure 2:**
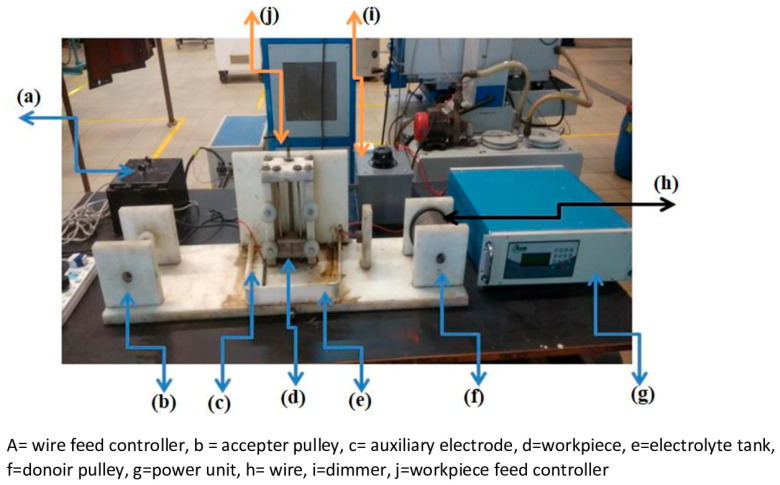
The TW-ECDM setup.

As discussed in the introduction section, owing to the higher hardness, nonconductivity, and higher melting point, the processing of quartz is difficult, and thus, quartz with the size of 75 × 25 × 2 mm was used as the workpiece material for this study [33]; some major properties of quartz material are given in Table 2. The TW-ECDM setup was designed and developed and the major units of the setup are as follows: passive (auxiliary) and the active (wire) electrodes, machining chamber, pulse DC power supply, wire feed controller, tank (contained the electrolyte), workpiece clamping, and the feed mechanism as shown in Figure 2.

**Figure 3:**
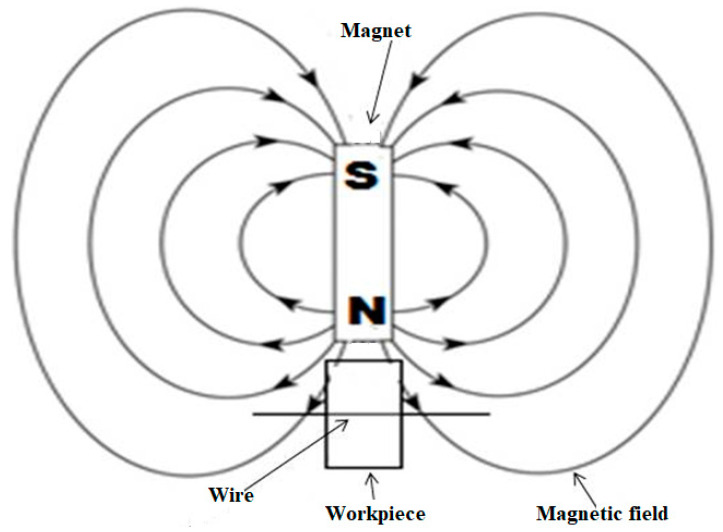
Positioning of the magnet and the workpiece [43].

A polymer-based material was utilized for the setup fabrication due to its exceptional scratch- and chemical-resistant nature. The size of the electrolyte tank was 120 × 100 × 40 mm. The passive (auxiliary) electrode was made of a 20 mm diameter graphite rod that was 80 mm in length. In the proposed study, a pulse mode DC power supply with a maximum voltage of 220 V and a current of 6 A was deployed. The positive and negative ends were connected to the anode and cathode, respectively. For guiding the wire, the pulley was made of Teflon material to maintain proper tension. A NaOH electrolyte was selected over KOH for better performance [36]. In the present work, a neodymium permanent magnet (disc type) was used to produce magnetic fields and the properties of the magnet are presented in Table 3. The workpiece and magnet were kept at a right angle to each other to obtain a better magnetic effect, as depicted in Figure 3.

For the analysis of the experimental results, MINITAB-17 software (Gandhinagar, Gujarat, India) was used to evaluate the performance of the process. An L_9_ orthogonal array (OA) was used to consider the variable parameters of voltage, electrolyte concentration, and wire feed (speed). Three response parameters, namely, the material removal rate (MRR), length of cut (LOC), and surface roughness (R_a_) were considered.

### Initial Trials

The conditions for the initial trials are summarized in Table 4.

Based on the initial trials, the available literature, and experimental restrictions, the range of input process parameters was selected to be wire speed: 0–15 m/min, electrolyte concentration: 25 to 35 (g/L), and voltage: 30–40 V. During the experiment, three levels of each input process parameter were selected, which are shown in Table 5. In addition to the Taguchi method, analysis of variance (ANOVA) was used to measure the impact of each input process parameter on the MRR, LOC, and R_a_. It also indicated the relative contribution of each input process parameter on the response. In the Taguchi design, the signal and noise represent the desired mean value and the undesirable (i.e., standard deviation) value of the output characteristics, respectively. In the present study, the interactions between the input machining parameters were neglected.

*MRR* was calculated using Equation (1):

(1) $$MRR=\left(\frac{W_{b}-W_{a}}{t}\right)$$

where *W_b_* and *W_a_* represent the weight of the workpiece before and after the experiment (mg/min), respectively, and *t* is the total machining time (min), where each experiment was designed for 10 min of machining time.

A METTLER TOLEDO model was used to measure the weight of the workpiece. To measure the LOC, a scanning electron microscope was used. A surface roughness tester (model no-ISR-S400) was used to measure *R_a_*. Equations (2)–(4) show the improvement ratio calculations of *MRR*, *LOC*, and *R_a_*, respectively:

(2) $$MRRImprovement\left(\%\right)=\left(\frac{MRR_{wmf}-MRR_{womf}}{MRR_{womf}}\right)\times 100$$

where *MRR_wmf_* is the material removal rate with a magnetic field (MHD) and *MRR_womf_* is the material removal rate without a magnetic field (MHD);

(3) $$R_{a}Improvement\left(\%\right)=\left(\frac{R_{a}{}_{womf}-R_{a}{}_{wmf}}{R_{a}{}_{womf}}\right)\times 100$$

where *R_awmf_* is the surface roughness with a magnetic field (MHD) and *R_awomf_* is the surface roughness without a magnetic field (MHD);

(4) $$LOCImprovement\left(\%\right)=\left(\frac{LOC_{wmf}-LOC_{womf}}{LOC_{womf}}\right)\times 10$$

where *LOC_wmf_* is the length of cut with a magnetic field (MHD) and *LOC_womf_* is the length of cut without a magnetic field (MHD).

## 3. Experimental Results and Discussion

Table 5 shows the combinations of input parameters for the response parameters as per the selected L_9_ orthogonal array. In the current study, the signal-to-noise (*S*/*N*) ratio was calculated for MRR and LOC based on larger being better and for R_a_ based on smaller being better. The *S*/*N* ratio was evaluated as per Equation (5) [49,50]: The S/N ratio values shown in Table 6.

(5) $$\frac{S}{N}=-10\times \log\left(\text{mean square deviation}\right)$$

### 3.1. Response Parameters

All the measured response parameters: material removal rate (MRR), Length of cut and Surface roughness (R_a_) has been analyzed. 

#### 3.1.1. Material Removal Rate (MRR)

**Figure 4:**
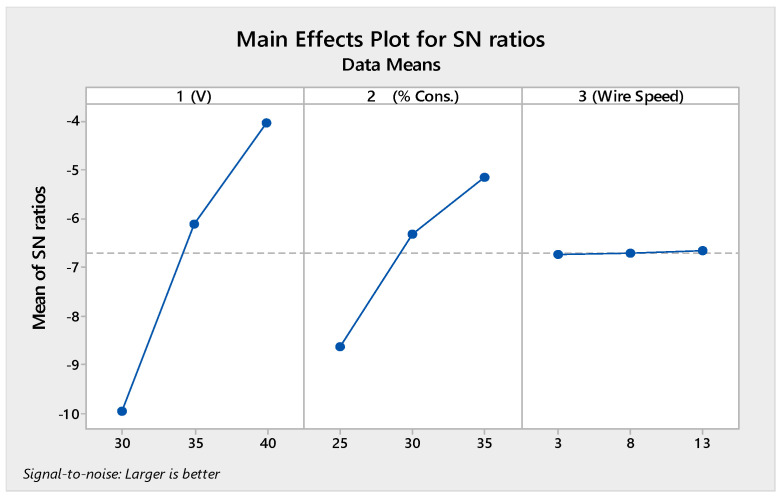
SN ratio plot for the MRR.

The best parametric set for all output parameters using the MHD method was found using L_9_ OA. Figure 4 demonstrates the signal-to-noise ratio plot for the MHD method for MRR. Table 7 shows the ANOVA table for the MRR response that was generated using the MHD method.

It is clear from Figure 4 and Table 7 that the voltage had the most important impact on the MRR, followed by the percentage of electrolyte concentration and wire speed. At low voltage, the sparking energy was lower, resulting in a lower MRR. Whenever the supply voltage increased, the rate of electrolysis increased, and therefore, high-intensity sparks were generated. Due to these high-intensity sparks, a larger crater was formed as compared to the lower-intensity sparks, resulting in a large quantity of material removed from the workpiece. With an increase in the concentration values, higher amounts of ions were available for electrolysis processes; hence, the rate of electrolysis was increased, and therefore, more bubbles were generated, which produced a stable highly dense gas film. This was responsible for the rise in MRR. The wire feed rate did not considerably affect the process performance. The optimal parametric set for the MRR was X_3_Y_3_Z_3_. The effect of increasing the machining voltage on the average maximum feed rate was greater than the effect of increasing the electrolyte concentration.

#### 3.1.2. Length of Cut (LOC)

**Figure 5:**
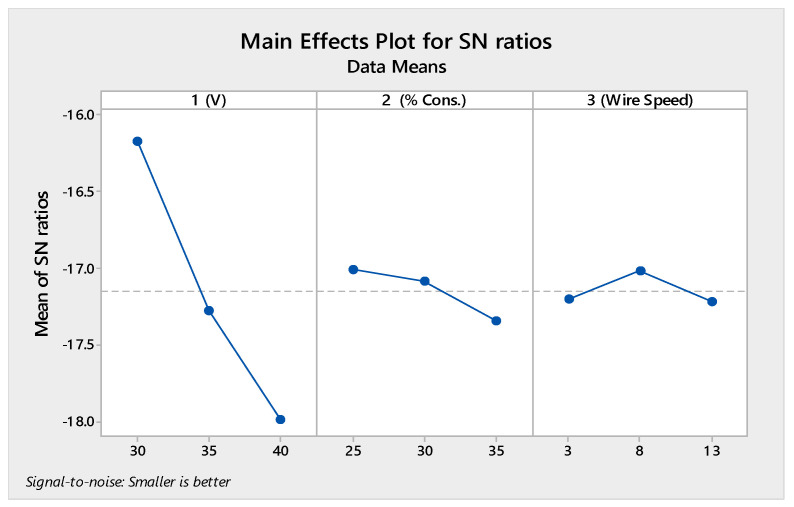
SN ratio graph for LOC.

Figure 5 displays the SN ratio graph for the MHD method and Table 8 illustrates the ANOVA table for the LOC created using the MHD method; it is evident that the voltage provided the greatest impact on the LOC, followed by the wire speed and the concentration percentage. As explained regarding the MRR, an increase in applied voltage increased the rate of electrolysis; hence, high-intensity sparks and the formation of a large crater were observed, which was responsible for the production of a large slit/cut along the cutting direction. The percentage of the electrolyte concentration and the wire speed negligibly affected the process. The increased electrolyte concentration with an increased wire speed resulted in a higher amount of ions available for the electrolysis process. Hence, increases in the rate of electrolysis and cutting speed were responsible for the increase in the LOC. The optimal parametric set for the LOC was X_3_Y_3_Z_3_.

#### 3.1.3. Surface Roughness (R_a_)

**Figure 6:**
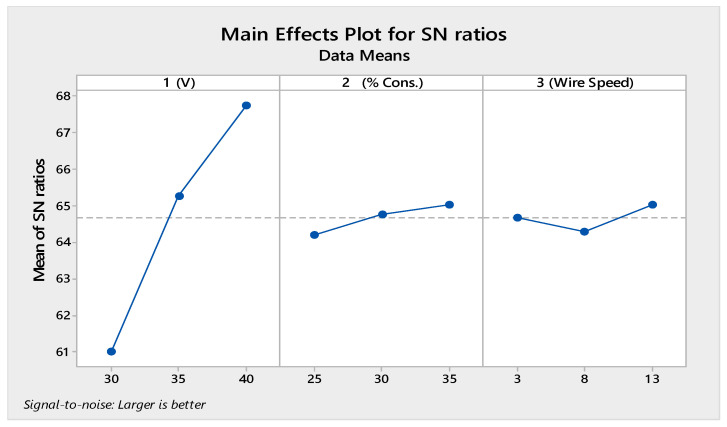
SN ratio graph for the LOC.

Figure 6 illustrates the SN ratio graph and Table 9 demonstrates the ANOVA for R_a_ when using the MHD approach; it is evident that the voltage had a significant effect on R_a_, followed by the electrolyte concentration and wire feed. As the voltage increased, the intensity of the sparks increased and side sparking also increased; therefore, a large crater was formed, which was responsible for generating coarse surfaces on the cutting area. As the wire speed increased, the tool electrode (wire) dragged the bubbles at a high speed away from the cutting area, resulting in an uneven spark that caused the rough surface. The optimal parametric set for R_a_ was X_1_Y_1_Z_2_.

### 3.2. Comparative Analysis

#### 3.2.1. Material Removal Rate Analysis

**Figure 7:**
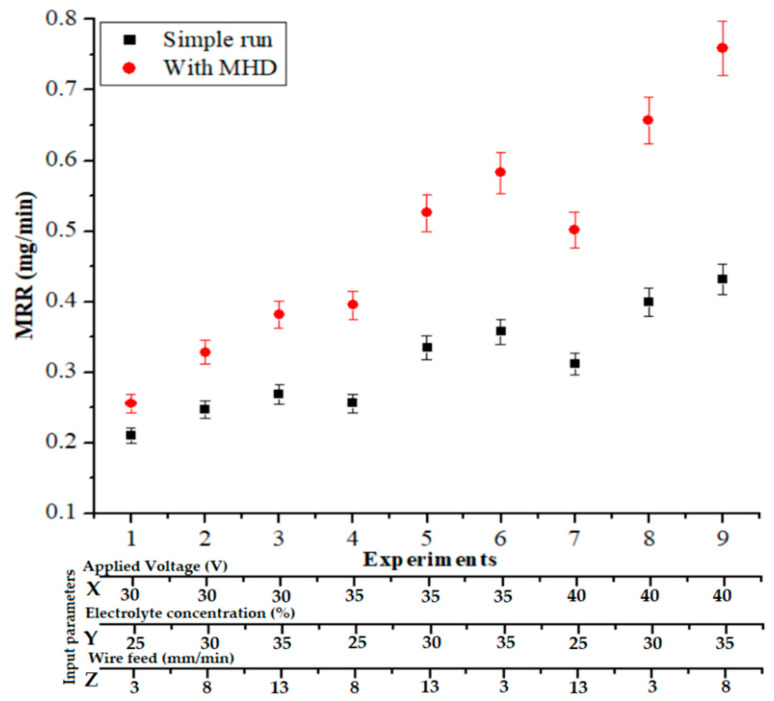
MRR with and without the MHD method being used.

Similar investigation conditions were also utilized during the analysis of the MRR with and without using the MHD approach. From Figure 7, it is clear that the MRR was significantly improved by 25.35% to 85.28% when using the MHD approach. In the MHD approach, the electrolyte distribution happened uniformly, allowing for more regular, steady, and high-intensity sparks to produce more hydrogen bubbles. However, in comparison with the approach without MHD, the electrolyte-enhanced stirring effect increased the MRR. Under the magnetic field at right angles to the electric field, a Lorentz force was produced, which was responsible for stirring the ions in the narrow gap between the tool wire and quartz. This could increase the void fraction and uncover the gas bubble’s surface. Furthermore, the electrolyte movement increased and removed the burrs from the quartz. Additionally, the coating on the wire increased the wire’s strength, prevented wire breakage, and increased the MRR and machining efficiency. The concentration of the solution generally affected both the dimensions of the channels created using chemical etching and the concentration of the spark energy, where the spark intensity affected the dimensions of the channels created by the process. Furthermore, when it was difficult to extend the electrolyte to the wire (tool), a downward force was exerted on the tool wire, which gently moved the tool toward the workpiece. At lower depths, the electrolyte easily reached the cutting area, where the removal of materials and continued chemical etching was more easily achieved. Due to the electric sparks striking the workpiece, which increased the local temperature and reduced the material viscosity, chemical corrosion occurred in a more favorable environment. In this area, the electric sparks were the main determinant of the material removal rate, and thus, it can be stated that machining was achieved in the electric discharge regime.

#### 3.2.2. Length of Cut Analysis

**Figure 8:**
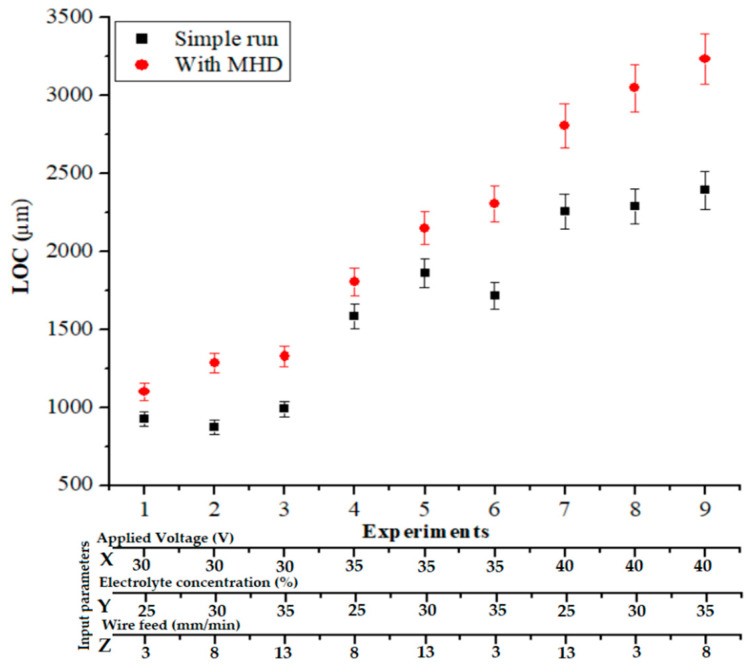
LOC (μm) with and without using the MHD method.

Similar investigation conditions were also utilized to analyze the LOC with and without using the MHD approach. Figure 8 shows an improvement in the LOC under the influence of a magnetic field by 7.04% to 48.86%. In the MHD method, the constant stirring caused a high-strength spark such that a large volume of material was removed from the quartz glass across the machining direction; due to the uniform electrolytic movement, this reduced overcut and increased the LOC. The electrolytic concentration affected the chemical etching regarding the magnitudes of the channels created and the concentration of the spark energy, where the spark intensity affected the dimensions of the microslits created by the process. Because the increase in voltage enhanced the sparks, this also improved the channel length. On the other hand, the presence of the sparks that jumped from the bottom of the tool to the workpiece played a major role in the material removal and determined the channel size. Increasing the voltage amplified the sparks and increased the length of the channel.

#### 3.2.3. Surface Roughness (Ra) Analysis

**Figure 9:**
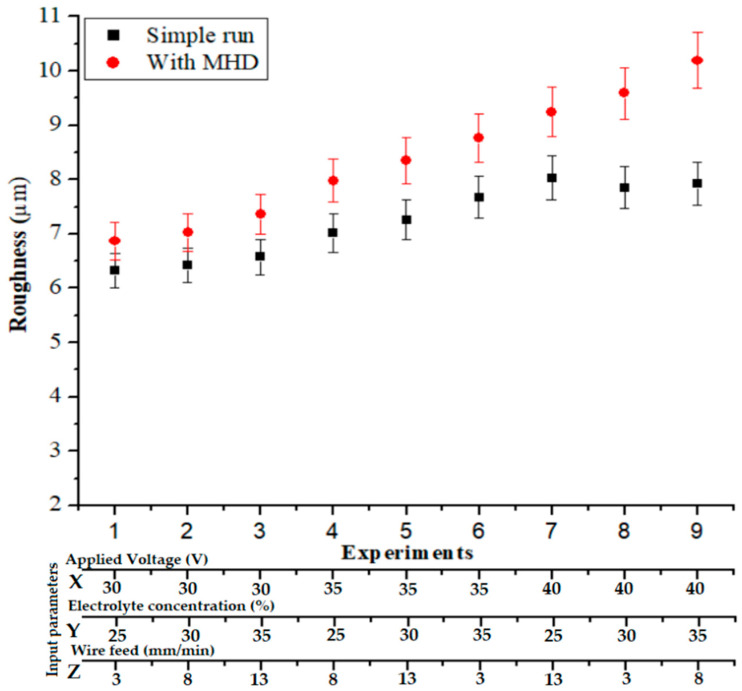
R_a_ (μm) with and without using the MHD method.

To analyze the effects of machining without and with using the MHD approach on R_a_, the same experimental conditions were used. From Figure 9, under the use of the MHD method, the average surface roughness decreased by 30.39%.to 9.06%. An increase in the applied voltage led to increased electrolysis formation, which caused high-strength sparks and, consequently, the generation of a bigger cavity that produced an irregular profile. The electrolyte stirring effect gave a finer surface profile than with the MHD method. Furthermore, debris was removed during the operation. During the MHD method, the electrolysis phenomenon was more steady and the sparking was higher at a higher level of electrolyte concentration; therefore, a fine average surface roughness with lower overcut was created. The more stable the gas film, the less time out of the whole machining process was devoted to forming the gas film, and the more time was devoted to the electric discharge, resulting in more spark energy. Increasing the spark energy led to more material removed per spark, resulting in a lower surface quality.

### 3.3. Mathematical Models and the Additivity Test

Multiple regression analysis was utilized to establish the correlations between the regulating process variables and the response features by adjusting the linear equation for the experimental values. The interaction of variable parameters was not considered due to L_9_ OA selection. Under two separate working conditions (with and without the MHD approach), the following mathematical equations could be used to model the machining of quartz glass with wire-ECDM, where Equations (6)–(8) show the mathematical models for the without-MHD run (simple run) for MRR, LOC, and R_a_, respectively:

(6) MRR=−0.4390+0.01390×(X)+0.00931×(Y)−0.00173×(Z)

(7) LOC=−3570+138.26×(X)+11.27×(Y)+5.93×(Z)

(8) $$R_{a}=-2.834+0.25907\times \left(X\right)+0.07377\times \left(Y\right)-0.00897\times \left(Z\right)$$

Equations (9)–(11) show the mathematical models for the with-MHD run for MRR, LOC, and R_a_, respectively:

(9) MRR=−1.171+0.03173×(X)+0.01902×(Y)−0.00286×(Z)

(10) LOC=−5264+179.2×(X)+38.49×(Y)−5.50×(Z)

(11) $$R_{a}=1.201+0.1494\times \left(X\right)+0.0267\times \left(Y\right)+0.0001\times \left(Z\right)$$

where *X* is the voltage (V), *Y* is the % electrolyte concentration (g/L), *Z* is the wire speed (m/min).

These mathematical models needed to be validated if it was to support an intermediary combination of process variables without a standard orthogonal array; hence, trials were run to check their validity. Table 10 represents the selected set of process variables and the response values. The error percentage demonstrates the slight deviation of the experimental value from the result predicted by the regression equations within limits. Furthermore, Table 11 shows the additivity test for the optimal parametric set.

### 3.4. Surface Profile Analysis Using SEM

**Figure 10:**
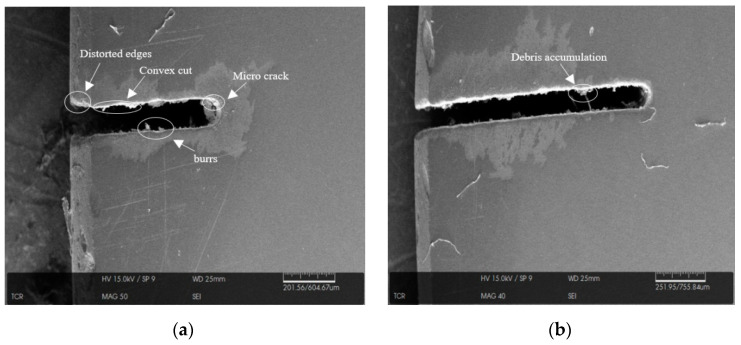
SEM images of slits produced (**a**) without using the MHD approach and (**b**) using the MHD approach.

Figure 10a,b shows the SEM images of the microslit produced on the quartz workpiece at 30 V, a 25% electrolyte concentration, and a 3 m/min wire speed for both the conventional and MHD-assisted processes. For the conventional process, the machined profile was found to have been rough and somewhat inconsistent. Figure 10b depicts the microslits in the workpiece under the application of MHD for the same parameter settings. The magnetic field assisted with eliminating the surface irregularities from the machined workpiece; therefore, the machined slits were found to be straighter with an enhanced cutting profile.

## 4. Conclusions

In this research, microslits with high aspect ratios were effectively fabricated on quartz via the MHD-assisted TW-ECDM process using a coated wire. A set of experiments with a lab-developed magnetically assisted TW-ECDM facility were conducted to study the magnetic field output effects. The effects of the voltage, percentage of concentration, and wire speed on the MRR, LOC, and R_a_ were presented. The following findings can be reported based on the experiments:

- The proposed magnet-assisted TW-ECDM process that used a zinc-coated brass wire was a useful method for microslit fabrication with a high aspect ratio on quartz material. The MHD method improved the electrolysis and reduced the burrs from the small gap between the workpiece and the tool wire.
- Due to the magnetic flux density over the current density, the electrolysis process was significantly improved. When using the MHD method, the MRR was increased from 25.35 to 85.28% in comparison to the non-MHD approach. Furthermore, the LOC improved by 7.04% to 48.86% for the MHD approach. 25
- The electrolytic concentration affected the chemical etching regarding both the magnitudes of the channels created and the concentration of the spark energy, where the spark intensity affected the dimensions of the microslits created by the process. Furthermore, when using the MHD method, a decrease in the surface roughness percentage was found to vary from 30.39% to 9.06% in comparison to the non-MHD method.
- Increasing the spark energy led to more sparking, resulting in a lower surface quality. The SEM image analysis showed better surface quality with the use of a coated wire during machining. The use of a coated wire minimized wire breakage and improved the effectiveness of machining, the MRR, the LOC, and the cutting speed. Moreover, electrolyte losses were reduced, resulting in lower costs and less environmental contaminants.

## Figures and Tables

**Table 1:** Types of wire (tool electrode) used TW-ECDM processes [20].

Ref.	Wire Material	Size of Wire (Diameter)	Workpiece Material	Observations
[5]	Stainless steel wire	0.25 mm	Optical glass, quartz, and ceramic (Al_2_O_3_*)*	Strong pulse DC supply was used to machine hard nonconducting materials.
[20]	Zinc-coated brass wire	0.15 mm	Quartz	Coated wire reduced the wire breakage and higher average surface roughness was observed because of craters, which could be minimized by providing flushing arrangements during the machining operation.
[23]	Zinc-coated brass wire	0.15 mm	Quartz	At a higher voltage and electrolyte concentration level, thin cracks with slim necking were observed at the opening of the machined surface. Furthermore, during machining, the debris became embedded at the workpiece and in the machining zone.
[28]	Textured Stainless-304 wire	0.20 mm	Borosilicate glass	Textured wire improved the localized electric field intensity. Lower ECD energy decreased the straightness of the microslits due to the edge-chipping effect.
[29]	Tungsten	0.05 mm	Quartz glass	Ultrasonic vibration refined the gas film’s thickness, improved the slit quality, and reduced the critical voltage.
[34]	Brass wire	0.15 mm	Quartz	By using a SiC powder-mixed titrated electrolytic flow, the surface quality and machining precision was improved.
[35]	Steel wire	0.070 and 0.090 mm	Al_2_O_3_ ceramic	Burrs, surface roughness, and slit expansion could be minimized by providing proper electrolyte flow.
[36]	Brass wire	0.25 mm	Glass and quartz	The reciprocating mechanism was beneficial for the machining of the irregular cross-section. Adding abrasive particles in the electrolyte reduced the slit expansion and improved the quality of the machined cut.
[37]	Brass wire	0.50 mm	Zirconate titanate	At higher voltages, large crack propagation and a slight workpiece melting phenomenon were observed.
[38]	Molybdenum wire	0.18 mm	Al_2_O_3_-particle-reinforced 6061 aluminum alloy	A longer pulsating supply reduced the MRR and improve the quality of surface and slit
[39]	Brass wire	0.25 mm	Borosilicate glass	Pulse DC power reduced the surafce roughness and Improve the material removal rate (MRR)
[40]	Brass wire	0.25 mm	Hylam-based composite	Under different experimental conditions, the surface finish, kerf width, and MRR improved.
[41]	Brass wire	0.25 mm	Borosilicate glass	Using a pulsed DC supply improved the MRR and reduced the kerf width via a selection of proper pulse on and off timings.
[42]	Brass wire	0.2 mm	Silica–epoxy nanocomposites	At higher voltages, the MRR and roughness were higher because of a decrease in the silica particle concentration.
[43]	Zinc-coated brass wire	0.15 mm	Quartz	The MRR increased from 21.47 to 75.82% and the surface roughness reduced from 22.25% to 7.93% under the application of the MHD approach with NaOH as an electrolyte.
[44]	Tungsten	0.1 mm	Glass	A rotating helical tool was proposed to construct a series of kerfs with a high aspect ratio. The gas film thickness could be increased using a proper electrolysis process.
[45]	Ni-coated brass wire	0.25 mm	SiC-reinforced z-pinned polymer matrix composites	Nonuniform cutting with a high diametrical overcut and lower material removal rate was observed.
[46]	Zn-coated brass wire		Quartz glass	Wire breakage occurred frequently and higher surface roughness, chips, and burrs were observed during machining of the surface.

**Table 2:** Selected properties of quartz glass [20].

Parameters	Values
Thermal expansion coefficient (20–320 °C)	5.11 × 10^−7^ cm/cm·°C
Density	2.2 × 10^3^ kg/m^3^
Hardness	5.5–6.5 Mohs scale (N/mm^2^)
Dimension	75(L) × 25(W) × 2(T) mm
Tensile strength	50 × 10^7^ Pa (N/m^2^)
Young’s modulus	7.2 × 10^10^ Pa

**Table 3:** Selected properties of the magnet.

Variant	Diameter	Length	Magnetic Field
Nd-Fe-B magnet (disc)	20 mm	10 mm	0.38 to 0.45 T (approx.) at the top surface

**Table 4:** Initial trial conditions.

Parameters	Range	Observation
Voltage range	<10 V	No electrolysis process was observed.
	10–20 V	Gas bubble formation was low, resulting in poor sparking.
	20–30 V	Low intensity of sparking with uniform electrolysis.
	30–40 V	Electrolysis rate was higher and the spark intensity was uniform and higher.
	Above 42 V	Unstable sparking led to frequent wire breakage.
Percentage of electrolyte concentration rage	<22%	Very narrow sparking was observed.
	22–40%	Sufficient and smooth sparking was observed.
	Above 40%	Uneven side sparking with high-strength sparking.
Wire speed (m/min)	<0–3 m/min	Wire breakage phenomenon with a coarse machined surface.
	Between 3–13 m/min	Wire breakage phenomenon was lessened and side sparking was reduced.
	Above 14 m/min	Cutting action was poor.

**Table 5:** Factors with their associated levels.

Factors	Input Parameters	Levels		
		1	2	3
X	Applied voltage (V)	30	35	40
Y	Electrolyte concentration (%)	25	30	35
Z	Wire feed (mm/min)	3	8	13

**Table 6:** S/N ratio for response parameters.

Exp. No.	Factor Levels			S/N Ratio		
	X	Y	Z	MRR	LOC	R_a_
1	1	1	1	−11.8453854	60.82982754	−16.023956
2	1	2	2	−9.67193703	60.71603662	−16.156110
3	1	3	3	−8.37238623	61.44080477	−16.35395
4	2	1	2	−8.06366128	64.10852656	−16.919315
5	2	2	3	−5.58524046	66.0414644	−17.211551
6	2	3	1	−4.69109964	65.60068046	−17.698171
7	3	1	3	−5.98938686	67.63446418	−18.092147
8	3	2	1	−3.65795191	67.56984763	−17.898499
9	3	3	2	−2.39287601	68.07781164	−17.979985

**Table 7:** ANOVA for the MRR for the MHD approach.

Factor	DOF	SS	MS	F-Value	*p*-Value	Contribution (%)
X	2	0.15184	0.07592	48.28	0.02	71.672
Y	2	0.055429	0.027714	17.62	0.054	26.162
Z	2	0.00144	0.00072	0.46	0.686	0.679
Error	2	0.003145	0.001573			0.014
Total	8	0.211862				98.529
Model Summary: R^2^—98.52%, R^2^Adj—94.06%						

**Table 8:** ANOVA for the LOC for the MHD approach.

Factor	DOF	SS	MS	F-Value	*p*-Value	Contribution (%)
X	2	4,821,541	2,410,770	296.83	0.003	95.034
Y	2	230,663	115,332	14.20	0.066	0.045
Z	2	5003	2501	0.31	0.765	0.098
Error	2	16,244	8122			0.320
Total	8	5,073,450				95.499
Model Summary: R^2^—99.68%, R^2^Adj—98.72%						

**Table 9:** ANOVA for the LOC for the MHD approach.

Factor	DOF	SS	MS	F-Value	*p*-Value	Contribution (%)
X	2	3.378	1.689	35.4	0.027	92.64934573
Y	2	0.119	0.059	1.2	0.444	3.276678725
Z	2	0.053	0.026	0.5	0.642	1.457459676
Error	2	0.095	0.047			2.616790133
Total	8	3.646				100.0002743
Model Summary: R^2^—97.38%; R^2^Adj—89.53%						

**Table 10:** Additivity test.

**Random Parametric Set for the Simple Runs**											
**TW-ECDM Parameters**			**MRR**			**R_a_**			**LOC**		
X	Y	Z	A	B	C	A	B	C	A	B	C
30	35	13	0.2684	0.2813	4.82	7.356	7.40344	0.644	991.15	1049.34	5.87
40	30	3	0.3992	0.3911	−2.06	9.586	9.71499	1.327	2289.13	2316.29	1.18
30	25	3	0.2105	0.2055	2.4	6.872	6.75544	1.69	925.61	877.34	5.5
**Random Parametric Set for the MHD Runs**											
**TW-ECDM Parameters**			**MRR**			**R_a_**			**LOC**		
X	Y	Z	A	B	C	A	B	C	A	B	C
30	35	13	0.3814	0.40942	6.843	6.572	6.6188	0.707	1329.15	1387.65	4.215
40	30	3	0.6563	0.66022	0.593	7.851	7.9783	1.595	3048.52	3042.2	−0.207
30	25	3	0.2557	0.24782	−3.18	6.327	6.3508	0.374	1100.25	1057.75	−4.017
where A signifies experimental values, B signifies mathematical equation values, and C signifies the error (%).											

**Table 11:** Additivity test for the optimal parametric set.

Optimal Parametric Set for the MHD Run		
MRR (mg/min)	R_a_ (μm)	LOC (μm)
0.8042	6.247	3415.29

## Data Availability

The data are available from the corresponding author upon request.
